# Obstructive Sleep Apnea and Smoking Increase the Risk of Cardiovascular Disease: Smoking Cessation Pharmacotherapy

**DOI:** 10.3390/jcm12247570

**Published:** 2023-12-08

**Authors:** Athanasia Pataka, Serafeim-Chrysovalantis Kotoulas, Aliki Karkala, Asterios Tzinas, George Kalamaras, Nectaria Kasnaki, Evdokia Sourla, Emiliza Stefanidou

**Affiliations:** 1 Respiratory Failure Unit, G. Papanikolaou Hospital Thessaloniki, Aristotle University of Thessaloniki, 57010 Thessaloniki, Greece; alikikarkala@gmail.com (A.K.); stergiostzinas@gmail.com (A.T.); kalamaras.giorgos@gmail.com (G.K.); kasnakinek@gmail.com (N.K.); evisou@yahoo.gr (E.S.); a_stefanidou@hotmail.com (E.S.); 2 ICU, Hippokratio General Hospital, 54643 Thessaloniki, Greece; akiskotoulas@hotmail.com

**Keywords:** obstructive sleep apnea, smoking cessation, cardiovascular, CVD, pharmacotherapy, nicotine replacement therapy, varenicline, bupropion, clonidine, nortriptyline, cytisine

## Abstract

Tobacco smoking has been a recognized risk factor for cardiovascular diseases (CVD). Smoking is a chronic relapsing disease and pharmacotherapy is a main component of smoking cessation. Obstructive sleep apnea (OSA) and smoking both increase the risk of CVD and are associated with significant morbidity and mortality. There are few existing data examining how pharmacological treatment, such as nicotine replacement therapy (NRT), bupropion, and varenicline, affect smokers suffering with OSA and especially their cardiovascular effects. The aim of this review was to evaluate the effects of smoking cessation pharmacotherapy on OSA with a special emphasis on the cardiovascular system. Results: Only small studies have assessed the effect of NRTs on OSA. Nicotine gum administration showed an improvement in respiratory events but with no permanent results. No specific studies were found on the effect of bupropion on OSA, and a limited number evaluated varenicline’s effects on sleep and specifically OSA. Varenicline administration in smokers suffering from OSA reduced the obstructive respiratory events, especially during REM. Studies on second-line medication (nortriptyline, clonidine, cytisine) are even more limited. There are still no studies evaluating the cardiovascular effects of smoking cessation medications on OSA patients. Conclusions: Sleep disturbances are common withdrawal effects during smoking cessation but could be also attributed to pharmacotherapy. Smokers should receive personalized treatment during their quitting attempts according to their individual needs and problems, including OSA. Future studies are needed in order to evaluate the efficacy and safety of smoking cessation medications in OSA patients.

## 1. Introduction

Tobacco smoking has been recognized to be a potent risk factor for cardiovascular diseases (CVD) such as strokes, coronary heart disease, peripheral vascular disease (PVD), atrial fibrillation, and sudden death [1]. It increases heart rate, blood pressure (BP), myocardial contractility, the rates of thrombosis, and worsens pre-existing heart failure. Smoking cessation is the most important step smokers can take in order to protect and enhance their cardiovascular health. Obstructive sleep apnea (OSA) is one of the most frequent causes of sleep disturbance and sleepiness. It is characterized by repetitive partial or complete obstruction of the upper airway that results in intermittent hypoxia and fragmentation of sleep [2]. Smoking and OSA both lead to increased risk of CVD and are associated with significant morbidity and mortality. Both conditions induce oxidative stress, inflammatory response, and endothelial dysfunction and adversely affect each other, leading to increased co-morbidity [3]. Studies have documented the relationship between sleep disorders (especially OSA) and CVD as leading to hypertension, stroke, arrhythmias, myocardial infarction, and heart failure [4]. Patients with severe OSA who smoke are at a greater cardiovascular risk than smokers with mild–moderate OSA and non-smokers [5].

Smokers continue to smoke as nicotine is very addictive. Nicotine addiction is considered to be a chronic disease with increased risk of relapse after withdrawal. The symptoms of withdrawal begin within a few hours after the last cigarette, may last for almost a month, and include the desire to smoke, attention deficits, irritability, augmented appetite, and sleep disturbances [6]. On the other hand, sleep disturbances are rather frequent during tobacco smoking, as nicotine leads to sleep fragmentation, reduced sleep efficiency (SE), increased sleep latency (SL), and decreased slow wave sleep (SWS) duration, resulting in increased daytime sleepiness [7,8,9]. 

The effect of OSA on nicotine addiction has not been studied extensively. There is a hypothesis that patients with untreated or undiagnosed OSA present higher nicotine addiction as nicotine’s stimulating effects help them to cope with sleepiness, loss of concentration, and even to lose weight [10,11]. Nicotine raises dopamine levels in nucleus accumbens, inducing the feeling of reward and arousal, and this enhances addiction, especially in chronically sleepy individuals [12]. Long-term hypoxia, as in the case of OSA, may further enhance the number of nicotine binding sites, contributing to smoking addiction. In accordance with that, the higher the number of nicotine receptors that are accessible, the greater the nicotine addiction [13]. In addition, smoking increases OSA severity by inducing upper airway inflammation, by affecting the upper airway neuromuscular function, and by altering sleep architecture and arousal mechanisms. There is a hypothesis that smoking cessation could improve OSA, but there is limited evidence to support this [3,10,14].

Smoking cessation has been associated with significant improvement in health and quality of life [15]. The use of pharmacological treatment, such as nicotine replacement therapy (NRT), bupropion, and varenicline, is recommended to increase the likelihood of quitting [16,17]. In the past, there have been concerns about the cardiovascular and neuropsychiatric safety of smoking cessation medications [18]. Subsequently, several studies in the general population and in specific patient groups with different pathologies (CVD, psychiatric and respiratory diseases) have concluded that they do not raise the risk of serious CVD and neuropsychiatric events [19,20]. Pharmacological treatment plays an important role in the success of smoking cessation [17], but there are few data to examine how it affects smokers with OSA.The aim of this review is to evaluate the effects of smoking cessation pharmacotherapy on OSA with a special emphasis on the cardiovascular effects of these medications.

## 2. Smoking Cessation Pharmacotherapy and OSA

Smoking is considered a chronic relapsing disease [16] and should be treated with different therapeutic interventions aimed at different aspects of nicotine addiction (physical, behavioral, psychological, and cognitive). The combination of behavioral support, education, and pharmacological treatment is the key to successful smoking cessation [17]. Clear but brief advice (3–5 min) provided by any healthcare professional significantly increases the motivation of a smoker to quit and increases abstinence rates [21].

Pharmacological treatment is an important component of smoking cessation interventions. First-line smoking cessation medications should be the first choice of clinicians as they have been approved by the European Medicines Agency (EMA) and are considered to be safe and effective in treating tobacco dependence. Nicotine replacement therapy (NRT), varenicline, and bupropion are the first-line therapies for smoking cessation [17]. Second-line medications include nortriptyline, clonidine, and cytisine that are not approved for smoking cessation in all European countries. They may be administered when first-line medications cannot be used [17]. Prolonging the duration of pharmacological treatment and a combination of different medications have been used in order to increase treatment efficacy [17,22,23].

Withdrawal symptoms are considered a main component in relapse and should be treated with the optimization of pharmacotherapy and the appropriate psychological support with counseling. Cravings and withdrawal symptoms should be evaluated at each follow-up visit of the smoker during the quitting process. During smoking cessation, sleep disturbances are frequent withdrawal symptoms and contribute to daytime symptoms such as irritability, memory loss, or concentration difficulties. Sleep problems and daytime somnolence negatively affect the ability of abstinent smokers to cope with everyday functioning, making relapse easier [24,25]. Patients suffering from OSA may have daytime somnolence due to sleep deprivation from the respiratory events. This may make their smoking cessation attempts more difficult. For that there is a need for the personalized evaluation of each smoker with the appropriate therapies according to their different needs during cessation. On the other hand, the different smoking cessation medications may affect sleep and sleep breathing events, especially in patients with OSA [14,24].

### 2.1. First-Line Medications

#### 2.1.1. Nicotine Replacement Therapy (NRT)

NRTs have been used for many decades for smoking cessation with significant efficacy in order to provide the nicotine of cigarettes without the damaging effects of smoke [26,27]. On the other hand, there are few and controversial studies on NRTs’ capacity to improve sleep disorders due to tobacco withdrawal [28,29]. There is evidence from self-reported questionnaires of abstinent smokers that NRTs increase sleep disruptions. On the other hand, in studies using objective data with sleep recordings, NRTs were not found to improve the subjective symptoms of sleep disturbances during cessation, whereas the objective signs were improved [30].

The effect of NRTs on the sleep architecture of non-smokers and former smokers is similar. In non-smokers, the application of a transdermal nicotine patch resulted in increased sleep stage 2, in rapid eye movement (REM) sleep reduction, and in REM sleep rebound during the nights that followed the discontinuation of the patch. In addition, no significant effects in sleep latency, sleep continuity, and total sleep time (TST) were found [31]. Furthermore, in smokers undergoing smoking cessation, the transdermal nicotine patch of 24 h duration appeared more effective than that of 16 h in sleep quality improvement by lowering microarousals and by increasing slow wave sleep (SWS) [32]. Both patches resulted in prolonged sleep latency and shorter TST, whereas only the 24 h patch improved NREM sleep, SWS, and arousals [32]. Studies using subjective sleep variables found that more sleep difficulties were reported in those who used nicotine patches, but the effect of the concomitant use of patches and smoking contributing to higher nicotine levels was not always evaluated [33]. 

As both withdrawal symptoms and nicotine administration may affect sleep, it is troublesome to discriminate between them. Withdrawal effects of nicotine addiction should not be underestimated as there are studies that found that sleep disorders continued with the use of nicotine patches, whereas in the placebo group they were even more frequent [11,24,33]. Additionally, side effects such as sleep disturbances are related to the duration of nicotine abstinence and the severity of nicotine dependence [33]. The reduction in plasma nicotine levels during the night may induce withdrawal symptoms, and especially heavy smokers may wake in order to smoke. This implies high nicotine dependence and increased risk of failure or relapse [28]. However, we should take into consideration that the heterogeneity of the methodology of the different studies, i.e., the different time and dose of NRTs, the additional use of conventional cigarettes, and the use of subjective or objective measures of sleep, limit the generalization of these results. 

There is evidence that nicotine has stimulant properties on the activity of the genioglossus muscle and on the ventilatory drive [7]. Further, there are studies with conflicting results evaluating the effect of nicotine for OSA treatment [11,12,14,34,35,36]. Nicotine gum (14 mg) use in OSA patients with different smoking histories resulted in the reduction in respiratory events (obstructive and mixed apneas) early at night during the first 2 h when the dilating properties of nicotine on the upper airway were efficient. However, this effect was not maintained as nicotine has a short half-life (2–4 h). For that, the respiratory events increased until the end of the night as upper airway resistance also increased. Further, sleep architecture, central apnea events, and end-tidal CO_2_ during wakefulness were not affected [34]. On the other hand, other studies where nicotine was administered by transdermal patches [35] or by tooth patches [36] did not find any significant alterations in respiratory events. In non-smoking OSA patients that received a transdermal nicotine patch (11 mg) for 12 h, versus a placebo, no positive effects on snoring or respiratory events [35] were found. However, a negative correlation between the mean duration of apneas and hypopneas and serum nicotine concentration was observed [35]. Sleep efficiency and TST were also reduced with the nicotine patch [35]. In addition, in another study where nicotine tooth patches in doses of 2 and 4 mg in OSA patients were used, no improvement of either the apnea hypopnea index (AHI) or sleep stages was found [36]. 

##### Cardiovascular Effects of NRTs

Nicotine’s action on the α4β2 nicotinic acetylcholinergic receptor (nAChR) enhances its reinforcing and addictive effects and on α3β4 nAChR mediates sympathetic neural stimulation. Due to this effect blood pressure, heart rate, and myocardial work increase. In addition, this may result in the constriction of coronary arteries and reduction in the myocardial blood supply. These effects may increase myocardial ischemia risk and the possibility of arrhythmias. It seems that smokers develop a degree of tolerance to these cardiovascular effects of nicotine. NRTs provide less plasma concentration of nicotine compared with cigarette smoking [37]. Several clinical studies have evaluated the cardiovascular effects of NRT, showing different results. Some studies found a significant increase in heart rate and systolic blood pressure following the use of NRTs [38,39,40,41] while others did not find any effects [42,43,44,45,46].

It has also been reported that nicotine patches (21 mg/24 h) increased the heart rate and blood pressure of non-smokers and normotensive smokers, but not of smokers with hypertension [44]. This could be explained by the fact that heavy smokers might develop tolerance to the effects of nicotine, resulting in no hemodynamic effects. In addition, a study assessing high cardiovascular risk patients did not show an association between nicotine patch use and first myocardial infarction [47]. Furthermore, a meta-analysis showed no increase in the risk of cardiovascular side effects such as hypertension, palpitations, arrhythmias, myocardial infarction, or stroke in patients using NRT versus those treated with a placebo [48], suggesting that NRTs are safe for smoking cessation.

Due to the aforementioned concerns on the cardiovascular safety of NRTs, some large clinical trials aimed to evaluate their safety, especially for patients with CVD [49,50,51]. These studies did not find increased cardiovascular risk in the group of patients using NRTs for smoking cessation. Cardiac arrest, myocardial infarction, death, and hospitalization due to arrhythmias, angina, or heart failure did not differ between the group using placebo and that using NRTs, although smoking cessation was more successfully achieved in the NRT group [52,53].

#### 2.1.2. Bupropion

Bupropion SR is a first-line medication for smoking cessation and the first non-nicotine therapy. Since 1989, it has been used as an anti-depressant and it has been observed that patients experienced smoking cessation unintentionally. For that, bupropion was evaluated for smoking cessation [54]. Bupropion is a weak norepinephrine–dopamine reuptake inhibitor but without significant antagonism at histaminic or muscarinic receptors. While it is not a classic stimulant, it may increase dopamine and norepinephrine resulting in non-specific stimulation [54,55,56]. Its main mechanism of action is by blocking the neuronal release of dopamine and noradrenaline and possibly by inhibiting anti-cholinergic nicotine receptors [55]. Bupropion’s efficacy for smoking cessation is independent from its anti-depressant action, as it has been proven effective also in non-depressive smokers. Bupropion reduces the severity of withdrawal symptoms such as depression and increased appetite and it has been found to almost double the abstinence ratio by reducing the severity of withdrawal syndrome in both sexes [56]. Bupropion is recommended for smoking cessation especially in smokers concerned about post-abstinence weight gain and for preventing smoking relapses. With the exception of one study [57] in smokers with chronic obstructive pulmonary disease (COPD), that expressed the hypothesis that bupropion could alter the ventilator response to hypoxia and hypercapnia, no other study has found similar effects [58]. 

As bupropion has been on the market for many years as an anti-depressant, its adverse effects have been well documented [58]. The most common adverse effects include headaches, oral dryness, and insomnia. More specifically, the short-acting formulations of bupropion that were administered late before sleep were associated with sleep disorders due to their alerting effects [59]. In order to avoid sleep problems such as insomnia, the first bupropion tablet is recommended in the early morning, so that the second tablet is administered at least four hours before sleep, early in the afternoon. If insomnia is considered a significant problem the dose may be reduced to 150 mg/day.

Moreover, unlike other anti-depressants, the use of bupropion in patients with depression may increase REM sleep and decrease REM latency [60]. A study has found that the differences in REM latency change after the administration of bupropion reflects the different response to the medication and the different response to treatment assessed by depression rating scales. Patients that showed an increase in REM latency after bupropion use responded better in the anti-depressive treatment compared with those who showed a decrease in REM latency that did not show anti-depressive response [61]. Further, it has been found that bupropion presents a relatively low risk of inducing REM sleep behavioral disorder [62] and restless legs syndrome [63,64].

Despite the administration of bupropion for many years as an anti-depressant and later for smoking cessation, there are few data concerning its effects in OSA patients. The co-existence of insomnia and OSA (Comorbid insomnia and sleep apnea, COMISA) is a rather common condition that has been under-recognized for several years. COMISA further impairs the quality of sleep and may cause problems in the diagnosis but also treatment. [65]. It has been observed that COMISA is linked with an increased risk of all-cause mortality and also higher rates of hypertension and cardiovascular disease (CVD) [66]. Insomnia is one of the most frequent side effects of bupropion; however, we did not find any studies evaluating the effects of bupropion on the sleep continuity of OSA patients and more specifically those suffering from COMISA. 

In addition, bupropion, unlike other anti-depressants, does not have REM suppressant effects and may increase REM [60]. Although OSA occurs during any sleep stage, the majority of respiratory events occur in REM, as during this stage of sleep there is reduced muscle tone in every muscle apart from the diaphragm. In addition, some patients present obstructive events only during REM sleep. For this reason, bupropion may affect the severity of OSA, especially in patients with predominantly REM OSA. However, this is a hypothesis as no studies are available yet. 

##### Cardiovascular Effects of Bupropion

The most important side effects of bupropion include the dose-related risk of seizures and hypertension reported even without the pre-existence of hypertension [54,55,56,57]. Precautions for these effects are included in the package label. Older studies did not find clinically important effects of bupropion on heart rate, blood pressure, conduction complications, or a higher risk of an AV block [67,68]. The use of bupropion SR for smoking cessation in patients with CVD has been also examined [69,70,71,72]. No significant effects on blood pressure were found at 12 weeks between bupropion and placebo and the adverse cardiovascular events were similar in both groups. However, the cardiovascular events were greater in the bupropion group after 12 months of observation, even though in the early post hoc analysis no significant differences were found between the cardiovascular events in patients who completed 30 days and 12 weeks of therapy [69]. Another study that evaluated the effect of bupropion in smokers hospitalized for acute coronary syndrome ended early due to a lack of efficacy, but did not find significant cardiovascular risk differences, even after one year of follow up [70]. Other studies that evaluated the cardiovascular effects of bupropion in the outpatient setting [71,72] did not find significant increase in blood pressure or heart rate, even in patients suffering from CVD. However, CVD patients that received bupropion reported more frequent adverse events such as palpitation and angina [71] and a small difference in heart rate occurred in those that received higher dose of bupropion SR (400 mg) [72]. 

#### 2.1.3. Varenicline

Varenicline is a first-line medication for smoking cessation and is one of the most effective ones as it has been found that it significantly increases smoking abstinence rates [73]. It binds with high selectivity and affinity with alpha4beta2 nAChRs (α4β2nAChRs). It also stimulates dopamine release [74]. Varenicline acts as a partial agonist reducing withdrawal symptoms, but also as an antagonist reducing the rewarding effects of smoking and facilitating smoking cessation. 

The most common side effects of varenicline include nausea, headaches, insomnia, and abnormal dreams. There is evidence that smokers that are treated with varenicline often report sleep disorders that decrease over time [24]. For example, insomnia symptoms peak during the first week of use and progressively decline after 2–12 weeks [75]. On the other hand, sleep disorders are a commonly reported symptom during smoking cessation and are considered a withdrawal symptom [11,24,76]. Furthermore, abnormal dreams and nightmares are also frequently reported as varenicline’s side effects [77,78]. Frequent awakenings and abnormal dreams but without significant alterations in sleep measures were reported in a study that evaluated sleep diaries after varenicline use [78]. Due to the stimulation of dopamine release from varenicline, amelioration of restless leg syndrome has been reported during smoking cessation attempts [79]. Rarely, in some cases, somnambulism and REM sleep disorders have been reported [77,79]. In the study of Savage et al. [77] that reviewed the original reports from WHO Global Individual Case Safety Reports Database, 27 reports were found about the adverse effects of varenicline. These included ten reports of aggressive activity during sleep and seven of other sleep-related harmful or potentially harmful activities (violent dreaming, nightmares, and other REM sleep behavior disorders, as well as NREM parasomnias such as somnambulism).

The data on the effect of varenicline on patients with OSA that smoke are rather limited. A study from our group showed that in smokers suffering from OSA, varenicline administration resulted in prolongation of sleep latency, of N2 and N3 sleep stage latency, in an increased arousal index, and in the reduction in AHI, especially during REM sleep [80]. Our main concern was the fact that it is difficult to differentiate between the adverse effects of varenicline or withdrawal symptoms per se. Unfortunately, due to the presence of N-nitroso-varenicline above the acceptable intake limits, varenicline has been recalled from European market over the last 2 years, limiting our studies to further explore its effects on sleep and sleep-disordered breathing [81]. 

##### Cardiovascular Effects of Varenicline

As varenicline binds selectively to α4β2nAChRs, its cardiovascular effects via the α3β4 nAChRs should be rather limited [82]. However, it has been found that varenicline binds to α7 homomeric nAChR, that may affect endothelial function and/or angiogenesis, contributing to cardiovascular adverse effects [83,84]. Early clinical trials including smokers with CVD did not find significantly higher rates of CV events as myocardial infarction and stroke compared to the placebo [85]. However, the US Food and Drug Administration (FDA) in 2011 mandated strengthened product warnings on the possible increased CV event risk in smokers with CVD [86]. In order to assess the neuropsychiatric adverse effects of bupropion and varenicline, the FDA and the EMA requested that their manufacturers conduct a randomized clinical trial (RCT) to evaluate their safety. The Evaluating Adverse Events in a Global Smoking Cessation Study (EAGLES) was extended in order to also evaluate possible cardiovascular events during and after smoking cessation treatment [19,20]. EAGLES was a large phase IV 24-week randomized double-blind, triple-dummy, placebo- and active-controlled trial in smokers with and without psychiatric disease and assessed the safety and efficacy of three first-line smoking cessation treatments: varenicline, bupropion with a tapering regimen with NRTs (control). This study concluded that there was no evidence of increased risk of serious cardiovascular adverse events with the use of smoking cessation pharmacotherapies [19,20]. 

In the meantime, several studies have reached mixed conclusions regarding varenicline’s safety [87,88,89,90,91,92]. An observational study reported a 34% increased risk of CVD hospitalizations and emergency department visits during varenicline use compared with controls, concluding that varenicline was associated with increased risk of cardiovascular but not neuropsychiatric events [92]. However, this study design was criticized for bias due to the inappropriate use of a self-controlled risk, due to reverse causality and measurement errors [93]. On the other hand, other studies on patients with acute coronary syndromes using varenicline did not report increased cardiovascular events but increased smoking abstinence compared with placebo [91,94]. In addition, more recent studies in a real-life setting have also reported minimal risk of cardiovascular side effects of first-line smoking cessation medication on cardiovascular events, heart rate, and blood pressure, further supporting their safety for patients with CVD [20,95,96].

### 2.2. Second-Line Medications

#### 2.2.1. Nortriptyline

Nortriptyline is a tricyclic anti-depressant that has been used as a second-line medication for smoking cessation [17,56,58]. The main indication of nortriptyline is as an antidepressant, but it has also been administered for chronic pain, i.e., orofacial pain, postherpetic neuralgia, and diabetic neuropathy (off-label). Nausea, headaches, constipation, dry mouth, sedation, and arrhythmia risk in patients suffering from CVD are its main side effects. Due to the potential side effects, the use of nortriptyline for smoking cessation is rather limited.

There are few studies evaluating the effects of nortriptyline on sleep and they are even more limited for patients with OSA. A study on elderly patients that were suffering from major depression found that nortriptyline decreased sleep apnea as it decreased REM sleep and increased phasic REM activity but did not have any effect on periodic limb movements during sleep [97].

##### Cardiovascular Effects of Nortriptyline

Clinical trials have shown that the use of nortriptyline increased heart rate and reduced heart rate variability, cardiac conduction, and cardio-respiratory coupling [98,99,100]. An animal study indicated that nortriptyline may affect QT prolongation [101]. On the contrary, subsequent studies evaluating elderly depressed patients [102] or non-depressed patients [103] supported its safety in patients with impaired cardiac function. Additionally, a cohort study evaluating anti-depressant medication did not find significant association with cardiovascular events or all-cause mortality risk [104].

#### 2.2.2. Clonidine

Clonidine is a second-line treatment for smoking cessation. It is an a2-adrenergic agonist that is indicated as an anti-hypertension treatment [105]. Its other off-label uses include drug withdrawal, certain pain conditions, flushing due to menopause, attention deficit hyperactivity disorder, and restless leg syndrome [102,106]. Clonidine is not approved worldwide as a medication for smoking cessation [106]. Clonidine’s most common adverse effects include postural hypotension, drowsiness, dry mouth, and fatigue and these side effects limit its use for smoking cessation.

The effects of clonidine on sleep and specifically on OSA have not been widely investigated. In a study including eight men suffering from OSA, clonidine hydrochloride was administered for ten days; this resulted in the reduction in respiratory events, as REM sleep was suppressed and REM sleep latency increased. However, during non-REM sleep, no changes were reported [107]. 

##### Cardiovascular Effects of Clonidine

Clonidine’s hemodynamic effects are mediated by both the heart and peripheral vascular system. Clonidine decreases heart rate and stroke volume, especially early in therapy, and reduces peripheral resistance, an effect that seems to persist even after the initial treatment period. Clonidine has a coronary vasodilating effect, proven beneficial for patients with coronary artery disease. Severe bradycardia is uncommon, even though clonidine reduces the heart rate. However, it should be used with caution in patients with AV conduction disease [108].

#### 2.2.3. Cytisine

Cytisine is nicotine receptor partial agonist that is used for smoking cessation and as a second-line medication in certain countries, especially in eastern European countries since 1960. It is an alkaloid that may be found in a number of plants. During the last years, the interest in the use cytisine for smoking cessation has increased due to its low cost [109]. Cytisine has similar characteristics to varenicline, as a partial agonist of alpha4beta2 nAChRs, preventing the binding of nicotine on these receptors and reducing cravings and symptoms of withdrawal [110,111]. The most common side effects of cytisine include nervousness, depression, vomiting, nausea, and sleep disorders [110]. Varenicline was more effective for smoking cessation compared with cytisine [112]. On the other hand, when cytisine was compared with NRTs, it was found to have superior effectiveness [111]. However, no studies on the possible effects of cytisine on patients with OSA were found.

##### Cardiovascular Effects of Cytisine

In one of the larger studies on cytisine [113], patients with a previous CVD such as severe atherosclerotic disease and those who have undergone percutaneous coronary interventions were excluded. In a recent study that evaluated the use of cytisine in patients with coronary artery disease 30 days after percutaneous coronary interventions found that it is a rather safe and promising treatment with no increase in cardiovascular complications. However, the compliance was rather low. Due to its low cost, cytisine may be beneficial for smoking cessation in patients with coronary artery disease. However further research is needed to confirm its efficacy and safety [114].

## 3. Discussion

According to recent and older studies [11,12,14,115,116,117,118], there is an association between smoking and increased OSA risk. A recent meta-analysis of 13 studies [116] demonstrated that AHI and sleepiness measured with the Epworth Sleepiness Scale (ESS) were significantly higher, whereas min SaO2 levels were lower in smokers compared with non-smokers. There was an association between severe OSA, pack-years, and smoking. Heavy smokers of more than 20 pack-years were at a higher OSA risk. Additionally, patients with more severe OSA exhibited a stronger association with smoking compared with those with mild or moderate disease [116].

In our opinion there is an association between successful smoking cessation and OSA, as untreated OSA may predispose one to increased nicotine addiction due to the stimulating effects of nicotine [14]. In addition, smoking increases OSA severity by inducing upper airway inflammation, affecting the upper airway neuromuscular function and by altering sleep architecture and arousal mechanisms [11,12]. Smoking cessation could improve OSA, but there is limited evidence. Sleep disturbances are included in the most common withdrawal effects during smoking cessation and are related to unsuccessful quitting attempts. We believe that smokers should receive personalized treatment according to their different individual needs and problems such as OSA. The effects of smoking cessation pharmacotherapy on the respiratory events and sleep architecture of patients with OSA are less studied and are presented in Table 1.

As for first-line treatments, only small studies have evaluated the effect of NRTs on OSA. Some of them found an improvement in respiratory events but the result was not permanent. No specific studies on the effect of bupropion on OSA patients were found. However, our hypothesis is that as bupropion increases REM sleep, it may increase OSA severity, especially during REM. A limited number of studies, including one from our group, evaluated the effect of varenicline on OSA. Varenicline administration in patients suffering from OSA that wished to stop smoking reduced the AHI, especially during REM sleep. Studies on second-line medications are even scarcer. Further investigations are required as it is difficult to differentiate the adverse effects of the medications and the withdrawal effects of smoking cessation.

Our review has several limitations. It is not a systematic review and it describes different studies with different aims and methods, so there is a bias risk. These studies include different types of population (i.e., smokers, with depression, with OSA, etc.), there is a limited number of participants in most of them and various outcomes. The different studies that were included in the current review evaluating smoking cessation medications on sleep and sleep disorders are summarized in Table 2.

## 4. Conclusions

It is critical to encourage smokers to quit, as smoking cessation is the most effective intervention to lower the risk of death and disability due to smoking-related chronic diseases. In Table 3, the main clinical recommendations for smoking cessation in OSA patients are summarized according to our opinion.

The impact of smoking and smoking cessation on sleep has been studied. On the one hand, there are limited studies on smoking cessation medications in patients with OSA and especially of their cardiovascular effects in this population. NRTs have been studied more extensively than the other first-line smoking cessation medications, such as bupropion and varenicline. The second-line medications, such as nortriptyline, clonidine, and cytosine, are even less studied. Furthermore, it is difficult to distinguish between the effects of smoking cessation per se and the adverse effects of smoking cessation pharmacotherapy. Future investigations with randomized controlled studies on the different pharmacotherapies are needed in order to eliminate these problems. Future studies are needed in order to evaluate the efficacy and safety of smoking cessation medications in OSA patients. OSA and cigarette smoking both increase CVD risk, so it is very important to help these patients to quit smoking effectively. OSA patients are at high CVD risk especially if left untreated. Smokers with CVD are most likely to quit following an acute event and smoking cessation treatments have been found to be safe for them [119]. Personalized treatment is important for every disease and smoking cessation should be performed in an individualized manner, taking into account the different problems of each patient, such as sleep disorders.

## Figures and Tables

**Table 1:** The effects of different smoking cessation pharmacological treatments on OSA.

**Smoking Cessation Pharmacotherapy**	**Effects on OSA**
First-line medications	
Nicotine replacement therapy (NRT)	The use of a transdermal patch: no significant improvement on respiratory events [35]. The use of a tooth patch: no significant improvement on respiratory events [36]. The use of nicotine gum: reduction in obstructive and mixed apneas during the first 2 h of sleep but the effect did not last as AHI increased during the end of the night [34].
Bupropion	No studies specifically on OSA patients. It increases insomnia symptoms [59] and may affect COMISA—no studies found. May increase respiratory events during REM as it increases REM sleep [60]—no studies found.
Varenicline	Prolongation of sleep latency. Increase N2 and N3 latency and arousal index. Reduction in AHI, especially during REM [80]. Insomnia, frequent awakenings, abnormal dreams are frequently reported [77,78].
Second-line medications	
Nortriptyline	No studies specifically on OSA patients. May decrease respiratory events as it reduces REM sleep [97]
Clonidine	No significant effect on AHI during non-REM sleep [107]. Reduction in respiratory events during REM, as it reduced REM sleep [107].
Cytisine	No studies specifically on OSA patients

OSA = obstructive sleep apnea, AHI = apnea hypopnea index, COMISA = comorbid insomnia and sleep apnea, REM = rapid eye movement.

**Table 2:** Details of the studies evaluating the different smoking cessation medications on sleep and sleep disorders.

**Type of Pharmacotherapy**	**Type of Study**	**Number Patients**	**Aim to Evaluate**	**Duration**	**Conclusions**
First-line					
NRT					
Jorenby et al. [26]	Multicentre comparative controlled clinical study	211	Intense counseling with either nicotine (21 mg) or placebo transdermal patches for withdrawal severity (using a nine-item daily self-report questionnaire, especially hunger and weight gain)	5-week study period	NRT 21 mg reduced craving for cigarettes, anxiety, irritability, appetite, and weight gain (1.85 versus 2.88 kg mean gain over 4 weeks in active and placebo groups, respectively)
Fredrickson et al. [27]	Open-label clinical trial	40	The safety and tolerability of 44 mg/day dose of transdermal NRT for smoking cessation in smokers ≥ 20 cigarettes/ day for 4 weeks followed by 4 weeks of 22 mg/day	4 weeks of each type of NRT	44 mg/day NRT patch was safe, tolerable, without significant adverse events. In 33% of subjects that received the 44 mg/day dose, sleep complaints were reported
Page et al. [29]	Blind clinical trial	15	The effect of 24-h transdermal NRT patches on sleep and dream mentation in smokers either 21 mg/24 h or 14 mg/24 h depending to smoking vs. control patch	1 night each intervention	More time awake, more arousals, and less REM sleep with NRT patch compared to placebo. Stages 1, 2, and SWS remained similar, suggesting nicotine effects specific to REM sleep and arousals. No general difference between dream report between NRT and placebo nights but more vivid dreams with NRTs
Wetter et al. [30]	Double-masked RCT	34	1. The impact of tobacco withdrawal on objectively assessed sleep parameters 2. If 24 h NRT aids or interferes with sleep during withdrawal Smokers received either active nicotine patches or placebo patches while quitting	PSG during two precessation and three postcessation nights	Tobacco withdrawal increases objectively assessed sleep disturbance (sleep fragmentation) NRT in postcessation improved sleep fragmentation, Stage 3, and Stage 4
Gillin et al. [31]	RCT	12	The dose-dependent, acute effects of NRT patch (7 and 14 mg) and placebo, applied 2 h before bedtime, on sleep and mood in non-smokers	PSG during two nights (NRT and recovery)	Compared with placebo patch, NRT associated with early morning awakening and reduced REM in a dose-dependent fashion. On the recovery night after the NRT, REM latency and stage 2 significantly reduced, whereas REM increased compared with the active patch. Mood and dreaming recall were not significantly affected
Staner et al. [32]	Open label two-period crossover RCT	20	The effects on sleep of 24 h (Nicopatch) or 16 h (Nicorette) NRT patches	48 h PSG from 12 pm to 7 am for two consecutive nights (baseline and treatment nights).	Compared to the 16 h NRT patch, the 24 h patch resulted in significantly less microarousals, a greater proportion of SWS, higher REM density, and REM beta activities
Gourlay et al. [33]	RCT double blind	629	The efficacy and safety of a repeat course of treatment with transdermal NRT for smoking cessation; 12 weeks with active NRT patches or placebo and brief counseling at monthly visits.	26-week follow up	Repeated treatment with NRT and brief counseling can improve low success rates. Difficulty in sleeping was reported by 24% on active treatment vs. 13.3% on placebo (*p* = 0.015)
For OSA					
Gothe et al. [34]	Clinical trial	8	The effect of NRT (nicotine gum total dose of 14 mg) on possible increase in upper airway muscle activity of OSA patients	One night	NRT reduced apneas (obstructive and mixed) during the first 2 h of sleep
Davilla et al. [35]	RCT crossover	20	The effect of NRT in the treatment of OSA, placebo, or an active patch that delivers 11 mg of nicotine over a 24 h period was applied	PSG one night	TST and SE decreased with NRT patch. Negative correlation between serum nicotine concentration and mean duration of respiratory events
Zevin et al. [36]	RCT	10	The effect of two doses of nicotine tooth patch, 2 mg and 4 mg, on OSA	PSG at baseline and during two treatments	No effect on AHI or on sleep stages even during the first 4 h when there were high levels of nicotine in saliva
Bupropion					
Gandotra et al. [59]	Observational retrospective study	30	The effect of different bupropion formulations on treatment-emergent insomnia in veterans with major depressive disorder	Baseline and at 4–6 weeks and 7–12 weeks after initiation of bupropion	SR and IR formulations were more often related with insomnia compared with the XL dosage form
Nofzinger et al. [60]	RCT	18	The effects of bupropion (*n* = 7), fluoxetine (*n* = 11), and cognitive behavior therapy on EEG sleep in depressed subjects	Pre- and post-treatment EEG sleep study	REM latency was reduced and REM% and REM time was increased with bupropion
Ott et al. [61]	RCT	20	The effect of bupropion SR (150 mg) single dose on sleep macroarchitecture and EEG changes in patients with unipolar major depressive disorder	2-night EEG session approximatey 1 week apart	Responders showed an increase in REM latency following bupropion challenge, whereas non-responders showed a decrease in REM latency
For Sleep Disorders					
McCall et al. [63]	Clinical trial observational	94	Whether OSA patients using SSRIs have more RRLMs than those taking bupropion or no anti-depressant. Comparison between groups bupropion (*n* = 32), an SSRI (*n* = 31), or no anti-depressant (*n* = 31)	One full-night diagnostic study or split-night study	Patients using SSRIs had significantly greater overall RRLM%, RRLM index, and PLMI relative to patients using bupropion and control patients
Bayard et al. [64]	RCT double blind	29	Whether 150 mg/day bupropion SR would improve the symptoms of RLS, or at least not exacerbate them (*n* = 29 bupropion vs. *n* = 31 controls)	6-week follow up	Bupropion was more effective than placebo in the treatment of RLS at 3 weeks; no significant difference at 6 weeks. Bupropion does not exacerbate RLS symptoms
Varenicline					
Savage et al. [77]	Original reports from WHO Global Individual Case Safety Reports Database	n/a	The adverse drug reaction reports of “abnormal sleep-related events” associated with varenicline	n/a	27 reports included 10 reports of aggressive activity during sleep and 7 of other sleep related harmful or potentially harmful activities (violent dreaming, nightmares, REM sleep behavior disorders, and NREM parasomnias somnambulism)
Polini et al. [78]	Clinical trial	38	Changes in sleep and dream measures by using a one-week sleep diary and collecting dream recall between varenicline (*n* = 25) and NRT (*n* = 13)	Baseline and 2 weeks follow up	Numerous dreams in the varenicline group may be related to lighter sleep due to increased number of awakenings
Romigni et al. [79]	Case report	1	Familial case of severe RLS resistant to treatment	Follow-up 12 weeks	Effective amelioration of RLS with varenicline
For OSA					
Pataka et al. [80]	Case-control study	30	Possible changes in PSG during varenicline treatment in healthy smokers (*n* = 16) and smokers with OSA (*n* = 14).	First PSG before varenicline while smoking and second 20–30 days during VAR administration and smoking cessation for at least 5 days	Varenicline treatment worsened sleep quality, as it prolonged SL, N2 and N3 latency, increased arousal index, and reduced SE. A marginal reduction in AHI was found in OSA patients, more significantly during REM
Second-line					
Nortriptyline					
Buysse et al. [97]	RCT double blind	31	The effects of nortriptyline and placebo on subjective and EEG sleep measures over 1 year of maintenance therapy and the likelihood of recurrence in elderly depressed patients	1 year	Nortriptyline acutely and persistently decreased REM, increased phasic REM activity, decreased sleep apnea, and had no effect on PLMs during sleep
Clonidine-OSA					
Issa FG [107]	Controlled clinical study	8	The effect of clonidine on OSA (10 days of clonidine 0.2 mg)	PSG in two control and two placebo nights	Clonidine reduced respiratory events, as REM sleep was suppressed and REM sleep latency increased. During NREM sleep, no changes were reported

NRT = nicotine replacement therapy, PSG = polysomnography, REM = rapid eye movements, SWS = slow wave sleep, OSA = obstructive sleep apnea, TST = total sleep time, SE = sleep efficiency, AHI = apnea hypopnea index, SR = sustained-release, IR = immediate-release, EEG = electroencephalogram, SSRIs = selective serotonin reuptake inhibitors, RRLMs = respiratory-related leg movements, PLMI = periodic limb movement index, RLS = restless legs syndrome, WHO = World Health Organization, SL = sleep latency.

**Table 3:** Clinical recommendations for smoking cessation in OSA patients.

A Multimodal Approach [16,17]: 1 + 2 + 3	Health Care Providers Should Assess Patient’s Nicotine Dependence to Understand the Chances for Success and Risk of Relapse (Five As: Ask, Advise, Assess, Assist, Arrange or AAR: Ask, Assist, Refer)		
Motivational strategies and behavior therapy (counseling)	OSA patients should consider smoking cessation as a part of their treatment Insomnia is a nicotine withdrawal symptom (consider CBT, short course benzodiazepines, melatonin) [24]		
2. Pharmacotherapy (first-line) Duration of therapy minimum of 12 weeks; may be prolonged [16,17] Consider combinations [17]: (no available studies for OSA patients) NRT long + short acting NRT + bupropion NRT + Varenicline	NRTs: Long-acting (nicotine patch), short-acting (nicotine gum, lozenge, inhaler, or nasal spray) according to nicotine dependence–number of cigarettes/day	Bupropion: * Especially in those with symptoms of depression or fatigue, while used it reduces postcessation weight gain Consider interactions with other medications, contraindications (i.e., history of seizures, brain tumors, etc.) Monitor for neuropsychiatric symptoms It increases insomnia, REM sleep (Table 1)	Varenicline: * Reduce dose in severe renal insufficiency Be cautious in patients with unstable psychiatric status, PTSD, suicidal ideation Monitor for neuropsychiatric symptoms Nausea, insomnia, abnormal (vivid, unusual, or strange) dreams (Table 1)
3. follow-up	Retreatment for smoking cessation as needed (counseling, other pharmacotherapy)	Follow up for OSA treatment (i.e., PAP adherence, MAD)	Take care for other issues (weight gain, exercise)

CBT = Cognitive Behavioral Treatment, NRT = Nicotine Replacement Treatment, PAP = Positive Airway Pressure, MAD = Mandibular Device, PTSD: posttraumatic stress disorder. * Gradual smoking reduction is an alternative.

## Data Availability

The study did not report any data.

## References

[1] Smoking and Tobacco Use. Centers for Disease Control and Prevention. http://www.cdc.gov/tobacco/data_statistics/fact_sheets/health_effects/effects_cig_smoking/index.htm.

[2] Jordan A.S., McSharry D.G., Malhotra A. (2013). Adult obstructive sleep apnoea. Lancet.

[3] Franklin K.A., Gíslason T., Omenaas E., Jõgi R., Jensen E.J., Lindberg E., Gunnbjörnsdóttir M., Nyström L., Laerum B.N., Björnsson E. (2004). The Influence of Active and Passive Smoking on Habitual Snoring. Am. J. Respir. Crit. Care Med..

[4] Drager L.F., McEvoy R.D., Barbe F., Lorenzi-Filho G., Redline S., INCOSACT Initiative (International Collaboration of Sleep Apnea Cardiovascular Trialists) (2017). Sleep Apnea and Cardiovascular Disease: Lessons from Recent Trials and Need for Team Science. Circulation.

[5] Lavie L., Lavie P. (2008). Smoking interacts with sleep apnea to increase cardiovascular risk. Sleep Med..

[6] Cummings K., Giovino G., Jaén C.R., Emrich L.J. (1985). Reports of smoking withdrawal symptoms over a 21 day period of abstinence. Addict. Behav..

[7] Jaehne A., Loessl B., Bárkai Z., Riemann D., Hornyak M. (2009). Effects of nicotine on sleep during consumption, withdrawal and replacement therapy. Sleep Med. Rev..

[8] Soldatos C.R., Kales J.D., Scharf M.B., Bixler E.O., Kales A. (1980). Cigarette smoking associated with sleep difficulty. Science.

[9] Zhang L., Samet J., Caffo B., Punjabi N.M. (2006). Cigarette smoking and nocturnal sleep architecture. Am. J. Epidemiol..

[10] Wetter D.W., Young T.B., Bidwell T.R., Badr M.S., Palta M. (1994). Smoking as a risk factor for sleep-disordered breathing. Arch. Intern. Med..

[11] Krishnan V., Dixon-Williams S., Thornton J.D. (2014). Where there is smoke there is sleep apnea: Exploring the relationship between smoking and sleep apnea. Chest.

[12] Taveira K.V.M., Kuntze M.M., Berretta F., De Souza B.D.M., Godolfim L.R., Demathe T., Canto G.D.L., Porporatti A.L. (2018). Association between obstructive sleep apnea and alcohol, caffeine and tobacco: A meta-analysis. J. Oral Rehabil..

[13] Collins A.C. (1990). Genetic influences on tobacco use: A review of human and animal studies. Int. J. Addict..

[14] Pataka A., Kotoulas S., Kalamaras G., Tzinas A., Grigoriou I., Kasnaki N., Argyropoulou P. (2022). Does Smoking Affect OSA? What about Smoking Cessation?. J. Clin. Med..

[15] Mayne S.L., Widome R., Carroll A.J., Schreiner P.J., Gordon-Larsen P., Jacobs D.R., Kershaw K.N. (2018). Longitudinal Associations of Smoke-Free Policies and Incident Cardiovascular Disease: CARDIA Study. Circulation.

[16] Steinberg M.B., Schmelzer A.C., Richardson D.L., Foulds J. (2008). The case for treating tobacco dependence as a chronic disease. Ann. Intern. Med..

[17] https://ensp.network/ensp-tdt-guidelines/.

[18] Mills E.J., Thorlund K., Eapen S., Wu P., Prochaska J.J. (2014). Cardiovascular events associated with smoking cessation pharmacotherapies: A network meta-analysis. Circulation.

[19] Anthenelli R.M., Benowitz N.L., West R., St Aubin L., McRae T., Lawrence D., Ascher J., Russ C., Krishen A., Evins A.E. (2016). Neuropsychiatric safety and efficacy of varenicline, bupropion, and nicotine patch in smokers with and without psychiatric disorders (EAGLES): A double-blind, randomised, placebo-controlled clinical trial. Lancet.

[20] Benowitz N.L., Pipe A., West R., Hays J.T., Tonstad S., McRae T., Lawrence D., St Aubin L., Anthenelli R.M. (2018). Cardiovascular Safety of Varenicline, Bupropion, and Nicotine Patch in Smokers: A Randomized Clinical Trial. JAMA Intern Med..

[21] Aveyard P., Begh R., Parsons A., West R. (2012). Brief opportunistic smoking cessation interventions: A systematic review and meta-analysisto compare advice to quit and offer of assistance. Addiction.

[22] Smith S.S., McCarthy D.E., Japuntich S.J., Christiansen B., Piper M.E., Jorenby D.E., Fraser D.L., Fiore M.C., Baker T.B., Jackson T.C. (2009). Comparative effectiveness of 5 smoking cessation pharmacotherapy in primary care clinics. Arch. Intern Med..

[23] Cahill K., Stevens S., Perera R., Lancaster T. (2013). Pharmacological interventions for smoking cessation: An overview and network meta-analysis. Cochrane Database Syst. Rev..

[24] Patterson F., Grandner M., Malone S.K., Rizzo A., Davey A., Edwards D.G. (2017). Sleep as a Target for Optimized Response to Smoking Cessation Treatment. Nicotine Tob. Res..

[25] Colrain I.M., Trinder J., Swan G.E. (2004). The impact on smoking cessation on objective and subjective markers of sleep: Review, synthesis and recommendations. Nicotine Tob. Res..

[26] Jorenby D.E., Hatsukami D.K., Smith S.S., Fiore M.C., Allen S., Jensen J., Baker T.B. (1996). Characterization of tobacco withdrawal symptoms: Transdermal nicotine reduces hunger and weight gain. Psychopharmacology.

[27] Fredrickson P.A., Lee G.M., Wingender L., Hurt R.D., Croghan I.T., Lauger G., Offord K.P., Gomez-Dahl L. (1995). High dose transdermal nicotine therapy for heavy smokers: Safety, tolerability and measurement of nicotine and cotinine levels. Psychopharmacology.

[28] Rieder A., Kunze U., Groman E., Kiefer I., Schoberberger R. (2001). Nocturnal sleep-disturbing nicotine craving: A newly described symptom of extreme nicotine dependence. Acta Med. Austriaca.

[29] Page F., Coleman G., Conduit R. (2006). The effect of transdermal nicotine patches on sleep and dreams. Physiol. Behav..

[30] Wetter D.W., Fiore M.C., Baker T.B., Young T.B. (1995). Tobacco withdrawal and nicotine replacement influence objective measures of sleep. J. Consult. Clin. Psychol..

[31] Gillin J.C., Lardon M., Ruiz C., Golshan S., Salin-Pascual R. (1994). Dose-dependent effects of transdermal nicotine on early morning awakening and rapid eye movement sleep time in non-smoking normal volunteers. J. Clin. Psychopharmacol..

[32] Staner L., Luthringer R., Dupont C., Aubin H., Lagrue G. (2006). Sleep effects of a 24-h versus a 16-h nicotine patch: A polysomnographic study during smoking cessation. Sleep Med..

[33] Gourlay S.G., Forbes A., Marriner T., Pethica D., McNeil J.J. (1995). Double blind trial of repeated treatment with nicotine patch for relapsed smokers. BMJ.

[34] Gothe B., Strohl K.P., Levin S., Cherniack N.S. (1985). Nicotine: A Different Approach to Treatment of Obstructive Sleep Apnea. Chest.

[35] Davila D.G., Hurt R.D., Offord K.P., Harris C.D., Shepard J.W. (1994). Acute effects of transdermal nicotine on sleep architecture, snoring, and sleep-disordered breathing in nonsmokers. Am. J. Respir. Crit. Care Med..

[36] Zevin S., Swed E., Cahan C. (2003). Clinical Effects of Locally Delivered Nicotine in Obstructive Sleep Apnea Syndrome. Am. J. Ther..

[37] Benowitz N.L., Gourlay S.G. (1997). Cardiovascular toxicity of nicotine: Implications for nicotine replacement therapy. J. Am. Coll. Cardiol..

[38] Mundal H.H., Hjemdahl P., Ghesdal K. (1995). Acute effects of low dose nicotine gum on platelet function in non-smoking hypertensive and normotensive men. Eur. J. Clin. Pharmacol..

[39] Fishbein L., O’Brien P., Hutson A., Theriaque D., Stacpoole P.W., Flotte T. (2000). Pharmacokinetics and pharmacodynamic effects of nicotine nasal spray devices on cardiovascular and pulmonary function. J. Investig. Med..

[40] Yugar-Toledo J.C., Ferreira-Melo S.E., Sabha M., Nogueira E.A., Coelho O.R., Colombo J.F.M., Irigoyen M.C., Moreno H. (2005). Blood pressure circadian rhythm and endothelial function in heavy smokers: Acute effects of transdermal nicotine. J. Clin. Hypertens..

[41] Najem B., Houssiere A., Pathak A., Janssen C., Lemogoum D., Xhaet O., Cuylits N., van de Borne P. (2006). Acute cardiovascular and sympathetic effects of nicotine replacement therapy. Hypertension.

[42] Khoury Z., Comans P., Keren A., Lerer T., Gavish A., Tzivoni D. (1996). Effects of transdermal nicotine patches on ambulatory ECK monitoring findings: A double-blind study in healthy smokers. Cardiovasc. Drugs Ther..

[43] Blann A.D., Steele C., McCollum C.N. (1997). The influence of smoking and of oral and transdermal nicotine on blood pressure, and haematology and coagulation indeces. Thromb. Haemost..

[44] Zevin S., Jacob P., Benowitz N.L. (1998). Dose-related cardiovascular and endocrine effects of transdermal nicotine. Clin. Pharmacol. Ther..

[45] Tanus-Santos J.E., Toledo J.C.Y., Cittadino M., Sabha M., Rocha J.C., Moreno H. (2001). Cardiovascular effects of transdermal nicotine in mildly hypertensive smokers. Am. J. Hypertens..

[46] Benowitz N.L., Hansson A., Jacob P. (2002). Cardiovascular effects of nasal and transdermal nicotine and cigarette smoking. Hypertension.

[47] Kimmel S.E., Berlin J.A., Miles C., Jaskowiak J., Carson J.L., Strom B.L. (2001). Risk of acute first myocardial infarction and use of nicotine patches in the general population. J. Am. Coll. Cardiol..

[48] Greenland S., Satterfield M.H., Lanes S.F. (1998). A meta-analysis to assess the incidence of adverse effects associated with the transdermal nicotine patch. Drug Safety..

[49] Working Group for the Study of Transdermal Nicotine in Patients with Coronary Artery Disease (1994). Nicotine replacement therapy for patients with coronary artery disease. Arch. Intern Med..

[50] Joesph A.M., Norman S.M., Ferry L.H., Prochazka A.V., Westman E.C., Steele B.G., Sherman S.E., Cleveland M., Antonuccio D.O., Hartman N. (1996). The safety of transdermal nicotine as an aid to smoking cessation in patients with cardiac disease. N. Engl. J. Med..

[51] Pack Q.R., Priya A., Lagu T.C., Pekow P.S., Atreya A., Rigotti N.A., Lindenauer P.K. (2018). Short-Term Safety of Nicotine Replacement in Smokers Hospitalized with Coronary Heart Disease. J. Am. Heart Assoc..

[52] Meine T.J., Patel M.R., Washam J.B., Pappas P.A., Jollis J.G. (2005). Safety and effectiveness of transdermal nicotine patch in smokers admitted with acute coronary syndromes. Am. J. Cardiol..

[53] Woold K.J., Zabad M.N., Post J.M., McNitt S., Williams G.C., Bisognano J.D. (2012). Effect of nicotine replacement therapy on cardiovascular outcomes after acute coronary syndromes. Am. J. Cardiol..

[54] Hays J.T., Hurt R.D., Decker P.A., Croghan I.T., Oxford K.P., Patten C.A. (2009). A randomized, controlled trial of Bupropion sustained release for preventing tobacco relapse in recovering alcoholics. Nicotine Tob. Res..

[55] Roddy E. (2004). Bupropion and other nonnicotine pharmacotherapies. BMJ.

[56] Hughes J.R., Stead L.F., Hartmann-Boyce J., Cahill K., Lancaster T. (2014). Antidepressants for smoking cessation. Cochrane Database Syst. Rev..

[57] GarcıaRıo F., Serrano S., Mediano O., Alonso A., Villamor J. (2001). Safety profile of Bupropion for chronic obstructive pulmonary disease. Lancet.

[58] Fiore M.C., Jaen C.R., Baker T.B., Mecklenburg R.E., Mermelstein R.J., Bailey W.C., Mullen P.D., Benowitz N.L., Orleans C.T., Curry S.C. (2008). Treating Tobacco use and dependence. Clinical Practice Guideline 2008 Update Rockville.

[59] Gandotra K., Jaskiw G.E., Williams S.G., Fuller M.A., Chen P., Wilson B., ElGhoul R., Konicki E., Strohl K.P. (2021). Development of Insomnia Associated with Different Formulations of Bupropion. Prim. Care Companion CNS Disord..

[60] Nofzinger E.A., Reynolds C.F., Thase M.E., Frank E., Jennings J.R., Fasiczka A.L., Sullivan L.R., Kupfer D.J. (1995). REM sleep enhancement by bupropion in depressed men. Am. J. Psychiatry.

[61] Ott G.E., Rao U., Nuccio I., Lin K.M., Poland R.E. (2002). Effect of bupropion-SR on REM sleep: Relationship to antidepressant response. Psychopharmacology.

[62] McCarter S.J., Howell M.J. (2016). Importance of Rapid Eye Movement Sleep Behavior Disorder to the Primary Care Physician. Mayo Clin. Proc..

[63] McCall C.A., Winkelman J.W. (2018). Respiratory-Related Leg Movements of Sleep Are Associated with Serotonergic Antidepressants but Not Bupropion. J. Clin. Sleep Med..

[64] Bayard M., Bailey B., Acharya D., Ambreen F., Duggal S., Kaur T., Roller K., Tudiver F., Rahman Z.U. (2011). Bupropion and Restless Legs Syndrome: A Randomized Controlled Trial. J. Am. Board Fam. Med..

[65] Sweetman A., Lack L., McEvoy D. (2019). Refining the measurement of insomnia in patients with obstructive sleep apnea. J. Clin. Sleep Med..

[66] Lechat B., Appleton S., Melaku Y.A., Hansen K., McEvoy R.D., Adams R., Catcheside P., Lack L., Eckert D.J., Sweetman A. (2022). Comorbid insomnia and sleep apnoea is associated with all-cause mortality. Eur. Respir. J..

[67] Roose S.P., Dalack G.W., Glassman A.H., Woodring S., Walsh B.T., Giardina E.G. (1991). Cardiovascular effects of bupropion in depressed patients with heart disease. Am. J. Psychiatry.

[68] Settle E.C., Stahl S.M., Batey S.R., Johnston J.A., Ascher J.A. (1999). Safety profile of sustained-release bupropion in depression: Results of three clinical trials. Clin Ther..

[69] Rigotti N.A., Thorndike A.N., Regan S., McKool K., Pasternak R.C., Chang Y., Swartz S., Torres-Finnerty N., Emmons K.M., Singer D.E. (2006). Bupropion for smokers hospitalized with acute cardiovascular disease. Am. J. Med..

[70] Planer D., Lev I., Elitzur Y., Sharon N., Ouzan E., Pugatsch T., Chasid M., Rom M., Lotan C. (2011). Bupropion for smoking cessation in patients with acute coronary syndrome. Arch. Intern Med..

[71] Tonstad S., Farsang C., Klaene G. (2003). Bupropion SR for smoking cessation in smokers with cardiovascular disease: A multicenter randomized study. Eur. Heart J..

[72] Thase M.E., Haight B.R., Johnson M.C. (2008). A randomized, double-blind, placebo-controlled study of the effects of sustained-release bupropion on blood pressure in individuals with mild untreated hypertension. J. Clin. Psychopharmacol..

[73] Tonstad S., Tønnesen P., Hajek P., Williams K.E., Billing C.B., Reeves K.R., Varenicline Phase 3 Study Group (2006). Effect of Maintenance Therapy with Varenicline on Smoking Cessation: A Randomized Controlled Trial. JAMA.

[74] Coe J.W., Brooks P.R., Vetelino M.G. (2005). An alpha4-beta2 nicotine receptor partial agonist for smoking cessation. J. Med. Chem..

[75] Foulds J., Russ C., Yu C.-R., Zou K.H., Galaznik A., Franzon M., Berg A., Hughes J.R. (2013). Effect of Varenicline on Individual Nicotine Withdrawal Symptoms: A Combined Analysis of Eight Randomized, Placebo-Controlled Trials. Nicotine Tob. Res..

[76] Htoo A., Talwar A., Feinsilver S., Greenberg H. (2004). Smoking and sleep disorders. Med. Clin. N. Am..

[77] Savage R.L., Zekarias A., Caduff-Janosa P. (2015). Varenicline and Abnormal Sleep Related Events. Sleep.

[78] Polini F., Principe R., Scarpelli S., Clementi F., De Gennaro L. (2016). Use of varenicline in smokeless tobacco cessation influences sleep quality and dream recall frequency but not dream affect. Sleep Med..

[79] Romigi A., Vitrani G. (2013). Improvement of Restless Legs Syndrome by Varenicline as Antismoking Treatment. J. Clin. Sleep Med..

[80] Pataka A., Frantzidis C., Kalamaras G., Gkivogkli P., Kotoulas S., Nday C., Chriskos P., Karagianni M., Styliadis C., Paraskevopoulos E. (2021). Varenicline administration for smoking cessation may reduce apnea hypopnea index in sleep apnea patients. Sleep Med..

[81] FDA Updates and Press Announcements on Nitrosamine in Varenicline (Chantix). https://www.fda.gov/drugs/drug-safety-and-availability/fda-updates-and-press-announcements-nitrosamine-varenicline-chantix.

[82] Rollema H., Chambers L.K., Coe J.W., Glowa J., Hurst R.S., Lebel L.A., Lu Y., Mansbach R.S., Mather R.J., Rovetti C.C. (2007). Pharmacological profile of the α4β2 nicotinic acetylcholine receptor partial agonist varenicline, an effective smoking cessation aid. Neuropharmacology.

[83] Mihalak K.B., Carroll F.I., Luetje C.W. (2006). Varenicline is a partial agonist at α4β2 and a full agonist at α7 neuronal nicotinic receptors. Mol. Pharmacol..

[84] Lee J., Cooke J.P. (2012). Nicotine and pathological angiogenesis. Life Sci..

[85] Rigotti N.A., Pipe A.L., Benowitz N.L., Arteaga C., Garza D., Tonstad S. (2010). Efficacy and safety of varenicline for smoking cessation in patients with cardiovascular disease: A randomized trial. Circulation.

[86] US Food and Drug Administration FDA Drug Safety Communication: Chantix (Varenicline) May Increase the Risk of Certain Cardiovascular Adverse Events in Patients with Cardiovascular Disease. http://www.fda.gov/Drugs/DrugSafety/ucm259161.htm.

[87] Prochaska J.J., Hilton J.F. (2012). Risk of cardiovascular serious adverse events associated with varenicline use for tobacco cessation: Systematic review and meta-analysis. BMJ.

[88] Sterling L.H., Windle S.B., Filion K.B., Touma L., Eisenberg M.J. (2016). Varenicline and adverse cardiovascular events: A systematic review and meta-analysis of randomized controlled trials. J. Am. Heart Assoc..

[89] Ware J.H., Vetrovec G.W., Miller A.B., Van Tosh A., Gaffney M., Yunis C., Arteaga C., Borer J.S. (2013). Cardiovascular safety of varenicline: Patient-level meta-analysis of randomized, blinded, placebo-controlled trials. Am. J. Ther..

[90] Kotz D., Viechtbauer W., Simpson C., van Schayck O.C., West R., Sheikh A. (2015). Cardiovascular and neuropsychiatric risks of varenicline: A retrospective cohort study. Lancet Respir Med..

[91] Eisenberg M.J., Windle S.B., Roy N., Old W., Grondin F.R., Bata I., Iskander A., Lauzon C., Srivastava N., Clarke A. (2016). EVITA Investigators. Varenicline for smoking cessation in hospitalized patients with acute coronary syndrome. Circulation.

[92] Gershon A.S., Campitelli M.A., Hawken S. (2018). Cardiovascular and neuropsychiatric events following varenicline use for smoking cessation. Am. J. Respir Crit. Care Med..

[93] Goulden R., Nguyen Q.D., Azoulay L. (2018). Risk of Bias in Study of Varenicline and Cardiovascular Outcomes. Am. J. Respir Crit Care Med..

[94] Windle S.B., Dehghani P., Roy N., Old W., Grondin F.R., Bata I., Iskander A., Lauzon C., Srivastava N., Clarke A. (2018). EVITA Investigators. Smoking abstinence 1 year after acute coronary syndrome: Follow-up from a randomized controlled trial of varenicline in patients admitted to hospital. CMAJ.

[95] Silva A.P., Scholz J., Abe T.O., Pinheiro G.G., Gaya P.V., Pereira A.C., Santos P.C. (2016). Influence of smoking cessation drugs on blood pressure and heart rate in patients with cardiovascular disease or high risk score: Real life setting. BMC Cardiovasc. Disord..

[96] Havard A., Choi S.K.Y., Pearson S.A., Chow C.K., Tran D.T., Filion K.B. (2021). Comparison of Cardiovascular Safety for Smoking Cessation Pharmacotherapies in a Population-Based Cohort in Australia. JAMA Netw. Open.

[97] Buysse D.J., Reynolds C.F., Hoch C.C., Houck P.R., Kupfer D.J., Mazumdar S., Frank E. (1996). Longitudinal effects of nortriptyline on EEG sleep and the likelihood of recurrence in elderly depressed patients. Neuropsychopharmacology.

[98] Roose S.P., Laghrissi-Thode F., Kennedy J.S., Nelson J.C., Bigger J.T., Pollock B.G., Gaffney A., Narayan M., Finkel M.S., McCafferty J. (1998). Comparison of paroxetine and nortriptyline in depressed patients with ischemic heart disease. JAMA.

[99] Bar K.J., Schuhmacher A., Hofels S., Schulz S., Voss A., Yeragani V.K., Maier W., Zobel A. (2010). Reduced cardio-respiratory coupling after treatment with nortriptyline in contrast to S-citalopram. J. Affect. Disord..

[100] Kiev A., Masco H.L., Wenger T.L., Johnston J.A., Batey S.R., Holloman L.C. (1994). The cardiovascular effects of bupropion and nortriptyline in depressed outpatients. Ann. Clin. Psychiatry.

[101] Jeon S.H., Jaekal J., Lee S.H., Choi B.H., Kim K.S., Jeong H.S., Han S.Y., Kim E.J. (2011). Effects of nortriptyline on QT prolongation: A safety pharmacology study. Hum. Exp. Toxicol..

[102] Thayssen P., Bjerre M., Kragh-Sorensen P., Moller M., Petersen O.L., Kristensen C.B., Gram L.F. (1981). Cardiovascular effect of imipramine and nortriptyline in elderly patients. Psychopharmacology.

[103] Giardina E.G., Johnson L.L., Vita J., Bigger J.T., Brem R.F. (1985). Effect of imipramine and nortriptyline on left ventricular function and blood pressure in patients treated for arrhythmias. Am. Heart J..

[104] Hamer M., Batty G.D., Seldenrijk A., Kivimaki M. (2011). Antidepressant medication use and future risk of cardiovascular disease: The Scottish Health Survey. Eur. Heart J..

[105] Sharma A., Gupta L. (2019). Clonidine aWonder Drug. Indian J. Anesth. Analg..

[106] Barua R.S., Rigotti N.A., Benowitz N.L., Cummings K.M., Jazayeri M.A., Morris P.B., Ratchford E.V., Sarna L., Stecker E.C., Wiggins B.S. (2018). ACC Expert Consensus Decision Pathway on Tobacco Cessation Treatment: A Report of the American College of Cardiology Task Force on Clinical Expert Consensus Documents. J. Am. Coll. Cardiol..

[107] Issa F.G. (1992). Effect of Clonidine in Obstructive Sleep Apnea. Am. Rev. Respir. Dis..

[108] Brest A.N. (1980). Hemodynamic and cardiac effects of clonidine. J. Cardiovasc. Pharmacol..

[109] Gotti C., Clementi F. (2021). Cytisine and cytisine derivatives. More than smoking cessation aids. Pharmacol. Res..

[110] Etter J.F. (2006). Cytisine for smoking cessation: A literature review and a meta-analysis. Arch. Intern Med..

[111] Walker N., Howe C., Bullen C., McRobbie H., Glover M., Parag V., Williman J., Veale R., Nosa V., Barnes J. (2011). Study protocol for a noninferiority trial of cytisine versus nicotine-replacement therapy in people motivated to quit smoking. BMC Public Health.

[112] Courtney R.J., McRobbie H., Tutka P., Weaver N.A., Petrie D., Mendelsohn C.P., Shakeshaft A., Talukder S., Macdonald C., Thomas D. (2021). Effect of Cytisine vs Varenicline on Smoking Cessation: A Randomized Clinical Trial. JAMA.

[113] West R., Zatonski W., Cedzynsk M., Lewandowska D., Pazik J., Aveyard P., Stapleton J. (2011). Placebo-controlled trial of cytosine for smoking cessation. N. Engl. J. Med..

[114] Ramotowski B., Budaj A. (2021). Is cytisine contraindicated in smoking patients with coronary artery disease after percutaneous coronary intervention?. Kardiol Pol..

[115] Yang Y., Wu J., Li S., Yu W., Zhu H., Wang Y., Li Y. (2023). Smoking, Coffee Consumption, Alcohol Intake, and Obstructive Sleep Apnea: A Mendelian Randomization Study. Curr. Neurovasc. Res..

[116] Zeng X., Ren Y., Wu K., Yang Q., Zhang S., Wang D., Luo Y., Zhang N. (2023). Association Between Smoking Behavior and Obstructive Sleep Apnea: A Systematic Review and Meta-Analysis. Nicotine Tob. Res..

[117] Deleanu O.C., Pocora D., Mihălcuţă S., Ulmeanu R., Zaharie A.M., Mihălţan F.D. (2016). Influence of smoking on sleep and obstructive sleep apnea syndrome. Pneumologia.

[118] Lin Y.N., Li Q.Y., Zhang X.J. (2012). Interaction between smoking and obstructive sleep apnea: Not just participants. Chin. Med. J..

[119] Tønnesen P., Lawrence D., Tonstad S. (2022). Medication-assisted quit rates in participants with smoking-related diseases in EAGLES: Post hoc analyses of a double-blind, randomized, placebo-controlled clinical trial. Tob. Induc. Dis..

